# HOSPITAL AT HOME PROGRAMS: CAREGIVER INSIGHTS ON SUCCESSES AND ENHANCEMENTS

**DOI:** 10.1093/geroni/igae098.3853

**Published:** 2024-12-31

**Authors:** Julia Hart, Brittni Howard, Jeannys Nnemnbeng, David Levine, Ksenia Gorbenko, Bruce Leff, Albert Siu, Abigail Baim-Lance

**Affiliations:** Icahn School of Medicine at Mount Sinai, New York, New York, United States; University of Massachusetts at Amherst, Amherst, Massachusetts, United States; Icahn School of Medicine at Mount Sinai, New York, New York, United States; Brigham and Women’s Hospital, Boston, Massachusetts, United States; Icahn School of Medicine at Mount Sinai, New York, New York, United States; Johns Hopkins Medicine, Baltimore, Maryland, United States; Icahn School of Medicine at Mount Sinai, New York, New York, United States; Veterans Health Administration, New York, New York, United States

## Abstract

Hospital at Home (HaH), an alternative to hospital-based care, continues to expand across the US. While the caregiver role is of great interest, little is known about their experiences. This is the first qualitative study, to our knowledge, examining caregiver experiences with HaH in a national sample. Beginning October 2023 (and ongoing), we conducted semi-structured interviews with caregivers via Zoom lasting 30-60 minutes, recruited through the HaH User’s Group, a national collaborative focused on implementation. This analysis uses rapid strategies including: systematic development of analysis templates, their synthesis to identify preliminary themes, and group consensus to ascertain meaning. To date, we completed 13 interviews with spouses, adult children, and grandchildren representing 5 programs (5 NE, 7 MW, 1 SE). 92% were female and 85% white. The mean age is 68 (range: 57-83). Program duration ranged from 3 days to 1 month. Overall, participants felt positive about HaH, reporting they would repeat the experience. Main benefits include: convenience, patient goal alignment, high quality care, and improved patient outcomes compared to brick and mortar experiences. Caregivers reported that providers were responsive, reliable and communicative. Yet, participants described lacking communication about caregiver roles and expectations, and desired additional information. Caregivers also reported needing more clarity about the timing of visits, tasks and procedures. They suggested that HaH may be more challenging to implement in contexts of greater caregiver strain or higher patient acuity. While caregivers generally reported positive HaH experiences, clearer and more explicit communication about the caregiver role could improve their experiences.

